# *In Silico* Analysis of *Arabidopsis thaliana* Peroxisomal 6-Phosphogluconate Dehydrogenase

**DOI:** 10.1155/2016/3482760

**Published:** 2016-02-29

**Authors:** Álvaro D. Fernández-Fernández, Francisco J. Corpas

**Affiliations:** Department of Biochemistry, Cell and Molecular Biology of Plants, Estación Experimental del Zaidín, CSIC, Apartado 419, 18080 Granada, Spain

## Abstract

NADPH, whose regeneration is critical for reductive biosynthesis and detoxification pathways, is an essential component in cell redox homeostasis. Peroxisomes are subcellular organelles with a complex biochemical machinery involved in signaling and stress processes by molecules such as hydrogen peroxide (H_2_O_2_) and nitric oxide (NO). NADPH is required by several peroxisomal enzymes involved in *β*-oxidation, NO, and glutathione (GSH) generation. Plants have various NADPH-generating dehydrogenases, one of which is 6-phosphogluconate dehydrogenase (6PGDH). Arabidopsis contains three* 6PGDH* genes that probably are encoded for cytosolic, chloroplastic/mitochondrial, and peroxisomal isozymes, although their specific functions remain largely unknown. This study focuses on the* in silico* analysis of the biochemical characteristics and gene expression of peroxisomal 6PGDH (p6PGDH) with the aim of understanding its potential function in the peroxisomal NADPH-recycling system. The data show that a group of plant 6PGDHs contains an archetypal type 1 peroxisomal targeting signal (PTS), while* in silico* gene expression analysis using affymetrix microarray data suggests that Arabidopsis p6PGDH appears to be mainly involved in xenobiotic response, growth, and developmental processes.

## 1. Introduction

Peroxisomes, which are present in almost all cells in eukaryotic organisms, are subcellular organelles delimited by a single membrane [1–3]. In biochemical terms, peroxisomes are characterized by a H_2_O_2_-producing flavin oxidase and hence their name, as well as a catalase enzyme which breaks down this H_2_O_2_ and is exclusive to this organelle [4, 5]. Plant peroxisomes are involved in important physiological metabolic pathways such as fatty acid *β*-oxidation (essential for germinating oilseeds, mobilizing reserve lipids, and providing carbohydrates), in oxidizing the products of photorespiration, cell signaling by reactive oxygen and nitrogen species (ROS and RNS, resp.), and phytohormone biosynthesis such as jasmonic acid (JA) and auxins [6–9]. In this last case, it has been shown that peroxisomal fatty acid *β*-oxidation machinery is responsible for *β*-oxidation of the carboxylic acid side chain of 12-oxophytoenoic acid (OPDA) in JA biosynthesis [9, 10]. On the other hand, indole-3-butyric acid (IBA) is a chain-elongated form of the active auxin indole-3-acetic acid (IAA) and based on genetic analysis and studies of IBA metabolism, IBA conversion to IAA occurs in peroxisomes [11–14]. In recent years, the identification of novel and unexpected peroxisomal proteins has raised questions concerning the potential functions of these organelles in this intensely studied field of cell biology. For example, the presence of nitric oxide (NO) in peroxisomes suggests that these organelles are a source of the signal molecule NO. This is supported by biochemical, molecular, and cellular approaches which indicate that plant peroxisomes contain a L-arginine-dependent nitric oxide synthase (NOS) activity which is strictly dependent on NADPH and requires calmodulin (CaM) and Ca^2+^ [15–17]. Moreover, this protein responsible to generate NO seems to be imported by a peroxisomal targeting signal type 2 (PTS-2) in a process that depends on the cytosolic receptor PEX7, CaM, and Ca^2+^ [18]. However, little is still known about many biological aspects of peroxisomes. Unlike chloroplasts and mitochondria, these organelles do not contain endogenous DNA, and all proteins are encoded by nuclear genes, synthesized in the cytoplasm, and imported into the peroxisomes [19–22]. While small molecules can access the peroxisomal matrix via passive transport [23], peroxisomal proteins contain a peroxisomal targeting signal (PTS) that enables them to be imported into the organelle. A group of proteins called peroxins (PEXs) are involved in this process, with a total of 22 peroxins having been identified in plants [3, 24]. To date, two main types of PTSs have been identified, each of which has its own receptor; the protein is then transferred to a translocation complex that mediates its transport across the peroxisome membrane. Most peroxisomal matrix proteins are targeted by a noncleavable tripeptide peroxisomal targeting signal 1 (PTS1) at the extreme C-terminus with the consensus sequence Ser-Lys-Leu (SKL). Other proteins are directed to peroxisomes by a cleavable nanopeptide peroxisomal targeting signal 2 (PTS2) at the N terminus bearing the sequence (R/K)-(L/V/I)-X_5_-(Q/H)-(L/A/I) [3].

**Figure 1:**
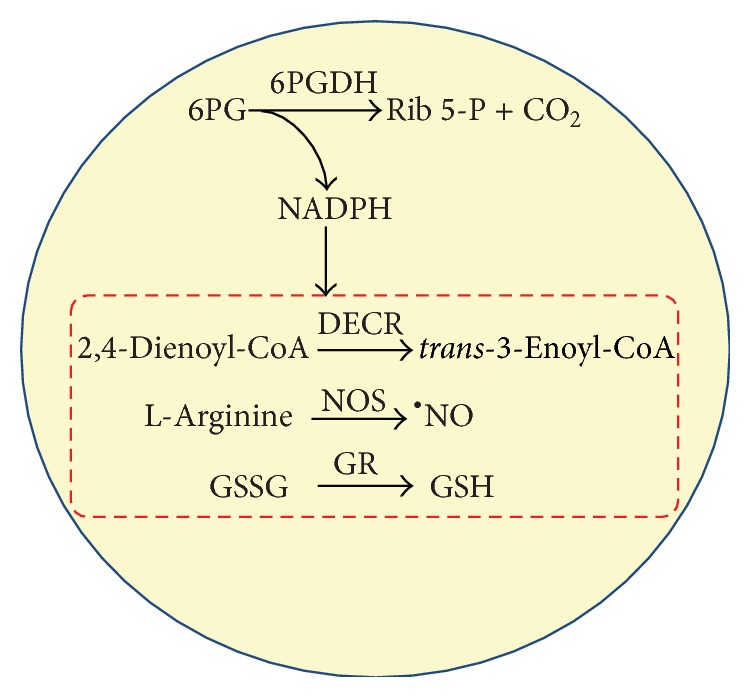
Functions of the endogenous NADPH in plant peroxisomes. NADPH is required for several enzymatic systems including the glutathione reductase (GR) to keep the level of reduced glutathione (GSH), the L-arginine-dependent nitric oxide synthase (NOS) which generates nitric oxide (NO), and the 2,4-dienoyl-CoA reductase (DECR) which is necessary for the degradation of fatty acids unsaturated on odd-numbered carbons. 6PGDH: 6-phosphogluconate dehydrogenase. 6PG: 6-phosphogluconate. Rib 5-P: ribulose 5-phosphate.

NADPH is an essential cofactor in many metabolic reactions. In plant peroxisomes, several enzymes activities are strictly dependent on the presence of NADPH such as L-arginine-dependent nitric oxide synthase (NOS) which facilitates NO production [1, 15], glutathione reductase (GR), which recycles reduced glutathione (GSH) from its oxidized form (GSSG) [1], and 2,4-dienoyl-CoA reductase (DECR), which participates in the degradation of unsaturated fatty enoyl-CoA esters and has double bonds in both even- and odd-numbered positions in peroxisomes [25, 26] (Figure 1). The different cell organelles are known to have multiple transporters in order to exchange metabolic intermediates [27–29]; however, as, to our knowledge, NADPH has no direct transport mechanism, each cell compartment must have its own NADPH-regenerating system. In higher plants, during the dark phase of photosynthesis and in nonphotosynthetic cells, NADPH is principally regenerated by a group of NADP-dehydrogenase enzymes including NADP-isocitrate dehydrogenase (NADP-ICDH), the NADP-malic enzyme (NADP-ME), glucose-6-phosphate dehydrogenase (G6PDH), and 6-phosphogluconate dehydrogenase (6PGDH), the latter two belonging to the oxidative pentose phosphate pathway (PPP) [30, 31]. Using electron microscopy immunogold labeling and biochemical techniques, previous studies have demonstrated the presence of several NADPH-generating dehydrogenases, such as NADP-ICDH [32, 33] and also G6PDH [34], in pea leaf peroxisomes. However, some biochemical [34] and molecular data [26] strongly suggest the potential localization of 6PGDH in peroxisomes. The present study therefore explores this possibility through an* in silico* analysis of plant 6PGDHs with particular emphasis on Arabidopsis 6PGDHs. The data reveal the presence of the archetypal PTS1 in a group of 6PGDHs, thus suggesting its peroxisomal localization, while affymetrix microarray data on the putative Arabidopsis p6PGDH suggest that it is involved in xenobiotic response, growth, and developmental processes.

## 2. Methods

### 2.1. Sequence Analysis, Database Searches, and Subcellular Localization Predictions

6PGDH protein sequences from completed genomes were retrieved from Plaza 3.0 (http://bioinformatics.psb.ugent.be/plaza/) [35]. Blast searches were carried out on the National Center for Biotechnology Information (NCBI) web site (http://www.ncbi.nlm.nih.gov/). Alignments were performed using CLUSTAL W2 (http://www.ebi.ac.uk/Tools/msa/clustalw2/). Localization predictions were made using WoLF (http://wolfpsort.org/) [36] and peroxisomal targeting signal type 1 (PTS1) Predictor (http://mendel.imp.ac.at/mendeljsp/sat/pts1/PTS1predictor.jsp) [37–39]. Molecular protein properties were estimated using the http://www.expasy.org/proteomics/protein_characterisation_and_function. Phylogenetic analyses were conducted using MEGA software version 6.0 (http://www.megasoftware.net/) [40]. The molecular properties of Arabidopsis 6PGDH isozymes based on their predicted amino acid sequences were used for the* in silico* predictions with the aid of http://www.expasy.org/tools/protparam.html.

Promoter analysis and identification of* cis*-regulatory elements were carried out according to the Plant Promoter 2.1 db program (http://ppdb.agr.gifu-u.ac.jp/ppdb/cgi-bin/index.cgi).

### 2.2. Gene Expression Analyses of Peroxisomal 6PGDH under Diverse Growth Conditions and Xenobiotics

Gene expression analyses under different conditions were carried out with the aid of the Gene Expression Omnibus (GEO) database (http://www.ncbi.nlm.nih.gov/geo/) [41] using Affymetrix Microarray Suite 5.0 (MAS5). The growth conditions selected were as follows. (i) For organ analysis,* Arabidopsis thaliana* plants were grown at a density of 4 plants per 12.7 cm square pot in either a growth chamber or green house set to 25°C by day and 20°C by night. Days were set to a 16 h photoperiod with 125 *μ*mol m^−2^ s^−1^ fluorescent irradiation. Expanding leaves were harvested 15 days after germination in midphotoperiod. The expanding upper 5 cm of the stem, with siliques and pedicels removed, was harvested 29 days after germination in midphotoperiod. Developed flowers and unopened buds were harvested 29 days after germination in midphotoperiod. (ii) For the sucrose effect on 4-day-old dark-grown seedlings,* Arabidopsis thaliana* ecotype Columbia was grown for 4 days in the dark at 23°C in multiwell plates containing half-strength Murashige Skoog (MS) medium without added sucrose. Samples were subsequently kept for 6 h under the same conditions with the addition of 90 mM sucrose (shaking, thus aerobic). (iii) For herbicide effect on 5-day-old seedlings, Arabidopsis seeds were grown on half-strength MS plates supplemented with 1% sucrose and grown at 21°C under continuous light (100 *μ*mol m^−2^ s^−1^) with and without 5 *μ*M norflurazon. (iv) For auxin treatments, Arabidopsis seedlings were grown for 10 d on 1x MS agar-solidified media under long-day conditions (16 : 8, white light and dark cycle). Seedlings were then transferred to 2 different liquid media containing either 0.1 *μ*M 2,4-D or 0.1 *μ*M 2,4-D plus 1 *μ*M brassinazole. After 8 h of treatment, the seedlings were blotted with paper towels to remove excess media and subjected to total RNA isolation.

## 3. Results and Discussion

### 3.1. *In Silico* Analysis of Plant 6PGDHs: Identification of Peroxisomal Targeting Signal 1 (PTS1)

At present, the information available in different plant databases has increased significantly and constitutes an unparalleled resource to provide complementary data for experimental studies [42]. The* in silico* analysis of putative peroxisomal 6PGDH aims to describe the most important properties of this protein in plants, with particular attention paid to* Arabidopsis thaliana*. This enzyme catalyzes the third and irreversible reaction of the pentose phosphate pathway (PPP) which conducts the oxidative decarboxylation of the free acid of 6-phosphogluconate to yield ribulose-5-phosphate, obtaining CO_2_ and NADPH. In higher plants, in comparison with the G6PDH enzyme that regulates the PPP, the 6PGDH enzyme has been much less studied and is assumed to have a cytosolic and chloroplastic localization [43, 44]. The phylogenetic analysis of 45 representative 6PGDH protein sequences from different organisms shows that plant 6PGDH proteins constitute a group clearly separated from the 6PGDH proteins of other organisms including prokaryote and eukaryote (see Supplemental Figure  1 in Supplementary Material available online at http://dx.doi.org/10.1155/2016/3482760). The representative 6PGDH sequences selected for this analysis are summarized in Supplemental Table  1.

**Figure 2:**
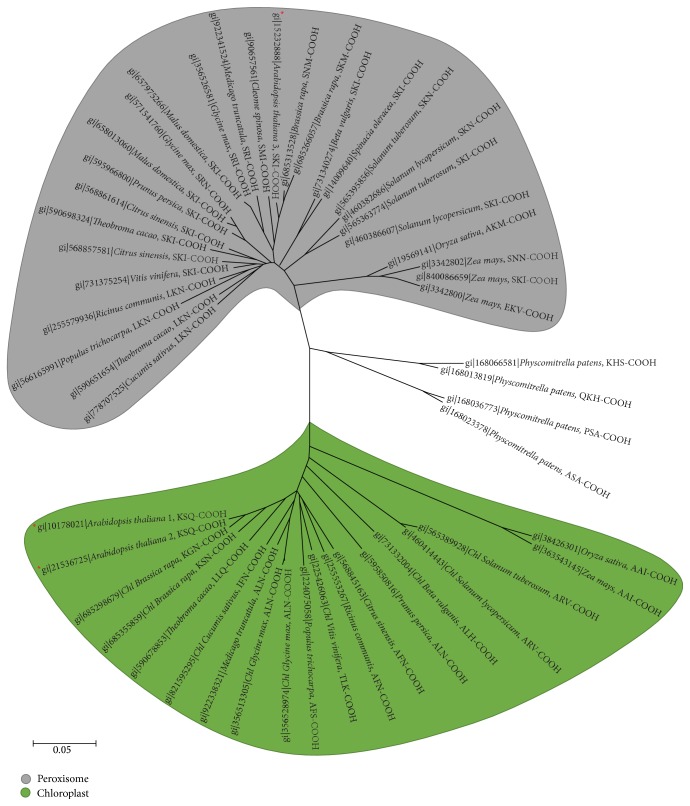
Evolutionary relationships of plant 6PGDHs. The evolutionary history was inferred using the Neighbor-Joining method. The optimal tree with the sum of branch length = 2,42428701 is shown. The tree is drawn to scale, with branch lengths in the same units as those of the evolutionary distances used to infer the phylogenetic tree. The evolutionary distances were computed using the Poisson correction method and are in the units of the number of amino acid substitutions per site. The rate variation among sites was modeled with a gamma distribution (shape parameter = 1). The analysis involved 51 amino acid sequences. All positions containing gaps and missing data were eliminated. There were a total of 416 positions in the final dataset. Evolutionary analyses were conducted in MEGA6 [30].

With the aim of gaining a deeper understanding of the subcellular localization of plant 6PGDHs, a phylogenetic analysis was carried out by exclusively using 51 plant 6PGDH protein sequences. Two main groups were found which appear to be located either in chloroplasts (32 sequences) or in peroxisomes (15 6PGDH sequences), with only four 6PGDH sequences belonging to these two main groups appearing to be located in the cytosol (Figure 2). In the peroxisome candidate group of 6PGDH sequences, a search of the main type I and type II peroxisomal targeting signals (PTSs) was carried out. This enabled us to identify a total of 10 6PGDH protein sequences which contain a PTS1 motif with the tripeptide SKI or SRI [22].  Table 1 summarizes some characteristics (number of amino acids, pI, and molecular mass) of these putative peroxisomal 6PGDH proteins of higher plants which have a putative type 1 peroxisomal targeting signal (PTS1). The tripeptides (SKI and SRI) identified belong to the canonical PTS1 sequence which is the tripeptide (S/A)-(K/R)-(L/M/I) at the extreme C-terminus. In general, these conserved tripeptides are highly abundant in peroxisomal matrix proteins [39], although other peroxisomal matrix proteins have noncanonical C-terminal tripeptides. The latter are much less conserved and generally occur in low-abundance peroxisomal matrix proteins [45, 46].

### 3.2. *In Silico* Analysis of Peroxisomal 6PGDH in* Arabidopsis thaliana*

At present, there are many genetic and biochemical information available on* Arabidopsis thaliana*. Therefore, this plant has become a powerful tool to study many aspects of higher plants [24]. Analysis of the Arabidopsis database shows that its genome contains three 6PGDH genes At5g41670, At3g02360, and At1g64190 coding for proteins BAB11473, AEE73797, and AAF24560, respectively. The alignment of the deduced amino acid sequence of the three Arabidopsis 6PGDHs indicates that the protein sequence of the three isozymes is highly conserved, with 75% similarity between BAB11473 and AEE73797 and 93% similarity between BAB11473 and AAF24560. In the sequence of deduced amino acids of the three Arabidopsis 6PGDHs, putative NADP binding sites with the GxG/VxxGxxxG consensus sequence and substrate binding sites with the L/I/V/M-x-D-x-x-G/AN/Q/S-KGTG-x-W sequence were identified (Supplemental Figure  2). These two sites are completely conserved throughout all 6PGDH sequences from others plant species.  Table 2 summarizes the principal molecular properties of each Arabidopsis 6PGDH isozyme based on the predicted amino acid sequences in each case. By using different subcellular prediction programs, it was found that 6PGDH can have different locations including chloroplasts, the cytosol, mitochondria, and peroxisomes, with subcellular localization showing the most significant differences among these 6PGDHs.

The gene encoding putative peroxisomal 6PGDH (At3g02360) has a total length of 2.25 kb, while its coordinates on chromosome 3 from* A. thaliana* are 481898 and 484147. The At3g02360 gene is transcribed in the nucleus using two possible mRNAs (NM_111103.5 and NM_180171.2) with lengths of 1,828 bp and 1,829 bp, respectively. Both mRNA molecules have an intron in the 5′-UTR region and two exons, one containing part of the 5′-UTR region and the other containing a small portion of the remaining 5′-UTR region, CDS, and 3′-UTR (Supplemental Figure  3 and Table 3). Promoter analysis enabled us to detect a variant of the TATA box at positions −565 and −553 (Table 3) as well as various regulatory elements identified in both transcripts (supplemental Figure  3) with the aid of the Plant Promoter 2.1 db program.

### 3.3. Gene Expression of Peroxisomal 6PGDH (p6PGDH)

**Figure 3:**
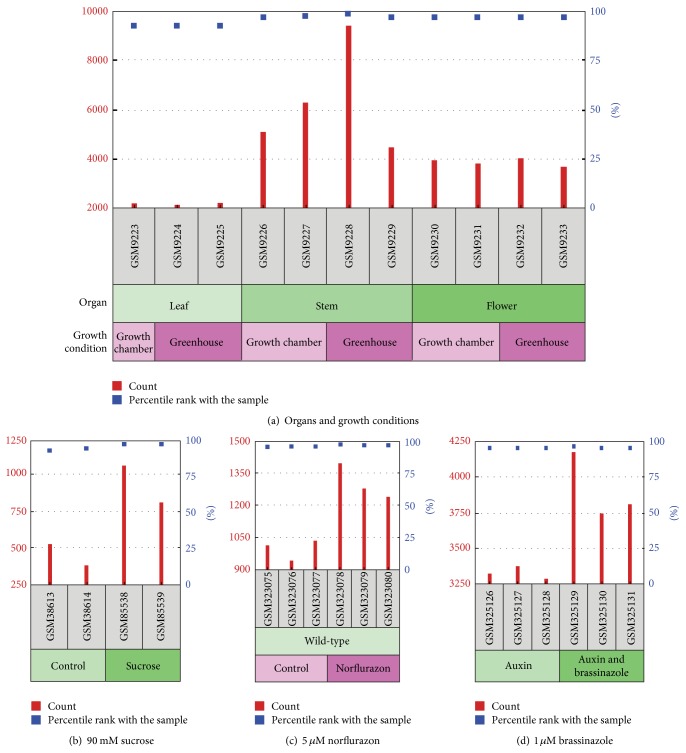
Peroxisomal* 6PGDH* gene (At3g02360) expression in* Arabidopsis thaliana* grown under different conditions. (a) Expression of* p6PGDH* in leaves of 15-day-old plants, stems and flowers of 29-day-old plants grown in either growth chamber or greenhouse conditions. (b) Effects of a 6 h long treatment with 90 mM sucrose to 4-day-old, dark-grown Arabidopsis seedlings. (c) Effects of 5 *μ*M norflurazon to 5-day-old, continuous light Arabidopsis seedlings. (d) Auxin effect on 10-day-old seedlings treated for 8 h either 0.1 *μ*M 2,4-D or 0.1 *μ*M 2,4-D plus 1 *μ*M brassinazole. Data were obtained from the Gene Expression Omnibus (GEO) database and analyzed using Affymetrix Microarray Suite 5.0 (MAS5). The original sample accessions (GSMxxx) are listed in the gray boxes along the bottom of the chart.

Figure 3 shows gene expression data for Arabidopsis* p6PGDH* under different growth conditions and is exposed to certain chemicals.  Figure 3(a) shows that, in adult plants,* p6PGDH* expression levels were highest in stems, followed by flowers and lowest in leaves, independently of the growth conditions in either the growth chamber or greenhouse. On the other hand, when Arabidopsis plants were grown under* in vitro* conditions and in the presence of 90 mM sucrose,* p6PGDH* gene expression was between 2-fold and 3-fold higher (Figure 3(b)), suggesting, as would be expected, that p6PGDH is involved in the carbon metabolism, as this enzyme is located in the oxidative part of the pentose phosphate pathway [43]. Additionally, when plants were exposed to the herbicide norflurazon (a carotenoid biosynthesis inhibitor), a similar 2-fold to 3-fold increase in* p6PGDH* gene expression was observed (Figure 3(c)). In the latter case, it is important to note that carotenoids have antioxidant properties that help to protect chlorophyll from oxidative damage mediated by ROS [47], as the absence of carotenoids facilitates chlorophyll destruction, which is essential for photosynthesis. These data are closely in line with the detoxification capacity of peroxisomes which would ameliorated the diminished antioxidant capacity of ROS to decompose chloroplasts damaged by this herbicide. This is explained by the fact that peroxisomes contain an important battery of antioxidant enzymes including catalase, superoxide dismutase, and all components of the ascorbate-glutathione cycle which requires NADPH to support the regeneration of GSH by GR [1]. Furthermore, the relative expression of* p6PGDH* was also higher in the presence of brassinazole (Figure 3(d)), a specific inhibitor of the biosynthesis of brassinosteroids, which are a class of phytohormones that play an essential role in plant growth and development processes, including promotion of stem elongation and cell division. All these data suggest that p6PGDH could be specifically involved in growth and development since its gene expression is clearly induced by molecules which block these processes. This is supported by a set of data which shows that sugar can promote hypocotyl elongation in Arabidopsis in darkness, a process which is largely dependent on brassinosteroids [48].

The reducing power (NADPH) generated by 6PGDH is known to be important, whose activity and gene expression can change depending on the type of stress and subcellular localization. For example, tobacco plants infected with potato virus Y show increased cytosolic and plastidic 6PGDH activity [49]. Similar behavior has been observed in pepper leaves, with an increase in total 6PGDH activity under cadmium [50] and low temperature [51] stress conditions. Similar behavior has been described with regard to* 6PGDH* gene expression. Thus, in rice (*Oryza sativa* L.) plants subjected to salt stress (150 mM NaCl), an increase in* 6PGDH* transcripts in stems has been reported [52]. A subsequent study, in which two genes encoding for chloroplastic and cytosolic* 6PGDH* rice plants, showed that both transcripts increased not only with salinity but also with other stresses such as drought, low temperature, and treatment with abscisic acid; however, the transcripts of* G6PDH*, the first enzyme of the oxidative portion of the pentose phosphate pathway, did not undergo any change in its cytosolic and chloroplastic isoforms [53]. Furthermore, there is evidence to show that this enzyme is also involved in other physiological processes; for example, in studies of the maturation of pepper fruits, when they change from the green to red phenotype, total 6PGDH activity increased by more than 50% [54]. In the case of tomato and spinach, three genes encoding cytosolic and chloroplastic isozymes have been reported [55, 56] also with respect to other plant species such as peas, corn, and tobacco [52, 57–59].

## 4. Conclusion

In summary, it is possible to conclude that there is a group of plant 6PGDH enzymes which contain an archetypal type 1 peroxisomal targeting signal. Given the capacity of this enzyme to generate NADPH,* in silico* analysis of peroxisomal 6PGDH in* Arabidopsis thaliana* suggests that it plays a prominent role during early seedling development in which peroxisomes perform the key function of metabolizing lipid reserves until the seedling begins to photosynthesize [60, 61], for which NADPH is required. Similarly, at this early seedling stage, the NADPH-dependent generation of nitric oxide in peroxisomes has also been demonstrated to be involved in root development [62]. Finally, the increase in* p6PGDH* in the presence of xenobiotics, such as norflurazon and brassinazole, is also closely in line with the induction of the antioxidant system present in plant peroxisomes as has been demonstrated with the pea leaf peroxisomes of plants exposed to 2,4-D [63]. Therefore, the present* in silico* analysis suggests the presence of a 6PGDH into peroxisomes which would contribute to the generation of NADPH within these organelles.

## Supplementary Material

Supplemental Figure 1. Evolutionary relationships of 45 protein sequences of 6PGDH from different taxa.
Supplemental Figure 2. Alignment of the three Arabidopsis 6PGDH protein sequences.
Supplemental Figura 3. Location of the regulatory elements of the promoter (PlantPromoterDB) of peroxisomal 6PGDH in *Arabidopsis thaliana*.
Supplemental Table 1. Characteristics of 6PGDH from different species used for the phylogenetic analysis.

## Figures and Tables

**Table 1:** Identification of 6PGDH proteins sequence of higher plants with a putative peroxisomal location for having a peroxisomal targeting signal type 1 (PTS1) on the C-terminal. The pI and MM values were calculated from their primary structure.

Plant specie	Aa length	PTS1	pI	Molecular Mass (kDa)	Accession number
*Arabidopsis thaliana*	486	-SKI	7.02	53.6	AEE73797.1
*Cleome spinosa*	485	-SMI	5.80	53.6	ABD96861.1
*Medicago sativa*	486	-SRI	5.33	53.7	AAB41553.1
*Oryza sativa*	480	-AKM	5.85	52.7	AJB98434.1
*Pinus sylvestris*	483	-SKI	6.31	53.3	ADP03057.1
*Pinus pinaster*	483	-SKI	6.74	53.2	ADP03318.1
*Spinacia oleracea*	483	-SKI	6.04	53.2	AAK51690.1
*Vitis vinifera*	438	-SKI	8.13	48.3	CAN67602.1
*Zea mays*	480	-SKI	6.09	52.7	ACN35899.1

**Table 2:** Genes encoding different isozymes of 6PGDH in *A. thaliana* and molecular properties based on their predicted amino acid sequence. The number of amino acids corresponds to the preprocessed protein and they were used for the *in silico* predictions using http://web.expasy.org/protparam/ Transit peptide (TP) or targeting signal (TS) length is given in amino acids, and molecular weight (MW) of the mature.

Properties	Locus		
	At5g41670	At3g02360	At1g64190
Protein accession number	BAB11473	AEE73797	AAF24560
Number of amino acids	487	486	487
Subunit size (Da)	53317.61	53577.18	53377.51
pI	5.62	7.02	5.34
Total number of negatively charged residues (Asp + Glu)	65	64	66
Total number of positively charged residues (Arg + Lys)	60	64	57
Stability index^*∗*^	23.69	26.86	27.60
*ε*_280_ (M^−1^ cm^−1^) (assuming all Cys residues are reduced)	65320	63830	63830
Aliphatic index^*∗∗*^	88.56	87.10	87.58
Grand average of hydropathicity (GRAVY) index^*∗∗∗*^	−0.278	−0.283	−0.272
Transit peptide (TP)/targeting signal (TS)	—	-SKI	—
Subcellular localization	Chloroplast/Cytosol	Peroxisome	Chloroplast/mitochondrion/Cytosol

^*∗*^A protein with a stability index smaller than 40 is predicted as being stable; with a value above 40 the protein is predicted as potentially unstable.

^*∗∗*^Aliphatic index of a protein is defined as the relative volume occupied by aliphatic side chains (Ala, Val, Ile, and Leu). A positive index indicates the increase of thermostability of globular proteins.

^*∗∗∗*^GRAVY (grand average of hydropathicity) index indicates the solubility of the proteins: positive GRAVY (hydrophobic), negative GRAVY (hydrophilic).

**Table 3:** Analysis of the 5′-UTR region At3G02360 gene coding for *Arabidopsis thaliana* peroxisomal 6PGDH. Inr.: initiator. TATA box: an octamer group related to TATA box. Y patch: an octamer group of the pyrimidine (Y) patch. REG: regulatory element group, an octamer group related to *cis*-regulatory elements. CDS: protein coding region. UTR: untranslated region.

Type	Sequence	Genome position			Position from initiation codon	
		Strand	Start	End	Start	End
TATA Box	TTGCTATATATCT	+	481933	481945	−565	−553
Y Patch	CTCTCCTCCTTC	+	481982	481993	−516	−505
Y Patch	TTCCTCT	+	482001	482008		−490
GA	None	None			None	
Inr.	None	None			None	
