# Supplementary material for: In Silico Analysis of Arabidopsis thaliana Peroxisomal 6-Phosphogluconate Dehydrogenase
Source: Scientifica (Cairo). 2016 Feb 29;2016:3482760. doi: 10.1155/2016/3482760 (PMC4789532; doi:10.1155/2016/3482760)
Supplement: Supplementary file 1 — Supplemental Figure 1. Evolutionary relationships of 45 protein sequences of 6PGDH from different taxa. Supplemental Figure 2. Alignment of the three Arabidopsis 6PGDH protein sequences. Supplemental Figura 3. Location of the regulatory elements of the promoter (PlantPromoterDB) of peroxisomal 6PGDH in Arabidopsis thaliana. Supplemental Table 1. Characteristics of 6PGDH from different species used for the phylogenetic analysis. [file 3482760.f1.pdf]

Supplemental Table 1. Characteristics of 6PGDH from different species used for the phylogenetic analysis. The pI and molecular weights were calculated by http://web.expasy. org/compute_pi.

Organism	Name	aa	pI	Mw	Accession	GI Number
Aggregatibacter actinomycetemcomitans	Aggac	484	5.68	53288.58	P70718	2492494
Agrobacterium fabrum	Agrfa	476	5.47	51265.75	AAK87317	15156614
Anopheles gambiae	Anoga	482	7.15	53142.03	XP_313091	158291584
Ascidia sydneiensis samea	Ascsy	483	6.68	53313.24	BAD98151	63003720
Arabidopsis thaliana	Arath 1	487	5.34	53377.51	NP_176601.1	15222639
Arabidopsis thaliana	Arath 3	486	7.02	53577.18	AEE73797	332640276
Arabidopsis thaliana	Arath 2	487	5.62	53317.61	NP_851113	30693852
Caenorhabditis elegans	Caeel	484	6.44	53196.12	NP_501998	17542558
Candida albicans	Canal	517	6.14	56924.03	BAA21690	2309076
Canis lupus	Canlu	483	6.65	53095.87	XP_535411	73950940
Ceratitis capitata	Cerca	481	6.72	52963.75	P41570	1168228
Chlamydophila pneumoniae	Chlpn	479	6.04	52938.80	BAA98568	8978732
Citrobacter freundii	Citfr	445	5.22	48832.36	AAC43814	540105
Cleome spinosa	Clesp	485	5.80	53582.29	ABD96861	90657561
Cucumis sativus	Cucsa	495	5.77	54084.35	ACM68927	222875454
Cunninghamella elegans	Cunel	485	5.84	53102.49	CAA76734	3152297
Drosophila melanogaster	Drome	481	6.04	52491.07	P41572	1168232
Escherichia coli K-12	Escco	468	5.04	51481.31	P00350	2506087
Haemophilus influenzae	Haein	484	5.32	53140.48	AAC22210	1573539
Homo sapiens	Homsa	483	6.80	53139.98	P52209	20981679
Lactococcus lactis	Lacla	472	4.91	52444.44	AAC12804	3033358
Laminaria digitata	Lamdi	530	6.19	57371.56	CAB61332	6453557
Medicago sativa	Medsa	486	5.33	53669.26	AAB41553	603221
Mesorhizobium loti	Meslo	475	6.29	50478.83	BAB50239	14023633
Mus musculus	Musmu	483	6.81	53247.20	NP_001074743	124486895
Neisseria meningitidis	Neime	482	5.26	52918.50	CAM07568	121051292
Neurospora crassa	Neucr	523	6.30	57247.39	XP_964959	758981115
Oryza sativa	Orysa 1	480	5.85	52721.21	NP_001056586	115465974
Oryza sativa	Orysa 2	508	6.09	54299.79	NP_001067912	115485537
Ovis aries	Oviar	483	7.59	52969.95	P00349	112844
Plasmodium falciparum	Plasfa	468	6.58	52994.01	XP_001348694	124809822
Rhodopirellula baltica	Rhoba	492	5.28	53999.48	CAD72844	32397538
Ricinus comunis	Ricco	495	6.25	54202.68	XP_002509902	255537671
Saccharomyces cerevisiae	Sacce	489	6.19	53543.25	NP_012053	398364913
Salmonella bongori	Salbo	445	5.35	48720.23	AAC43923	540191
Selaginella moellendorffii	Selmo	487	5.94	53210.68	XP_002966753	302766665
Schizosaccharomyces pombe	Schpo.	508	8.09	55629.70	BAA13823	1749530

Sinorhizobium meliloti	Sinme	476	5.92	50804.32	CAC46511	15074954
Spinacia oleracea	Spinacia	537	5.54	58293.21	AAK49897	13936693
Spinacia oleracea	Spinacia	483	6.04	53245.03	AAK51690	14009640
Synechococcus elongatus	Synechococcu s	471	4.98	51073.22	P21577	93141227
Synechocystis sp.	Synechocystis	482	5.11	52873.52	P52208	1703020
Thermotoga maritima	Thema	469	5.79	53018.85	AAD35523	4980945
Vitis vinifera	Vitis	438	8.13	48286.29	CAN67602	147795381
Zea mays	Zeama	482	6.24	52952.44	AAC27703	3342802

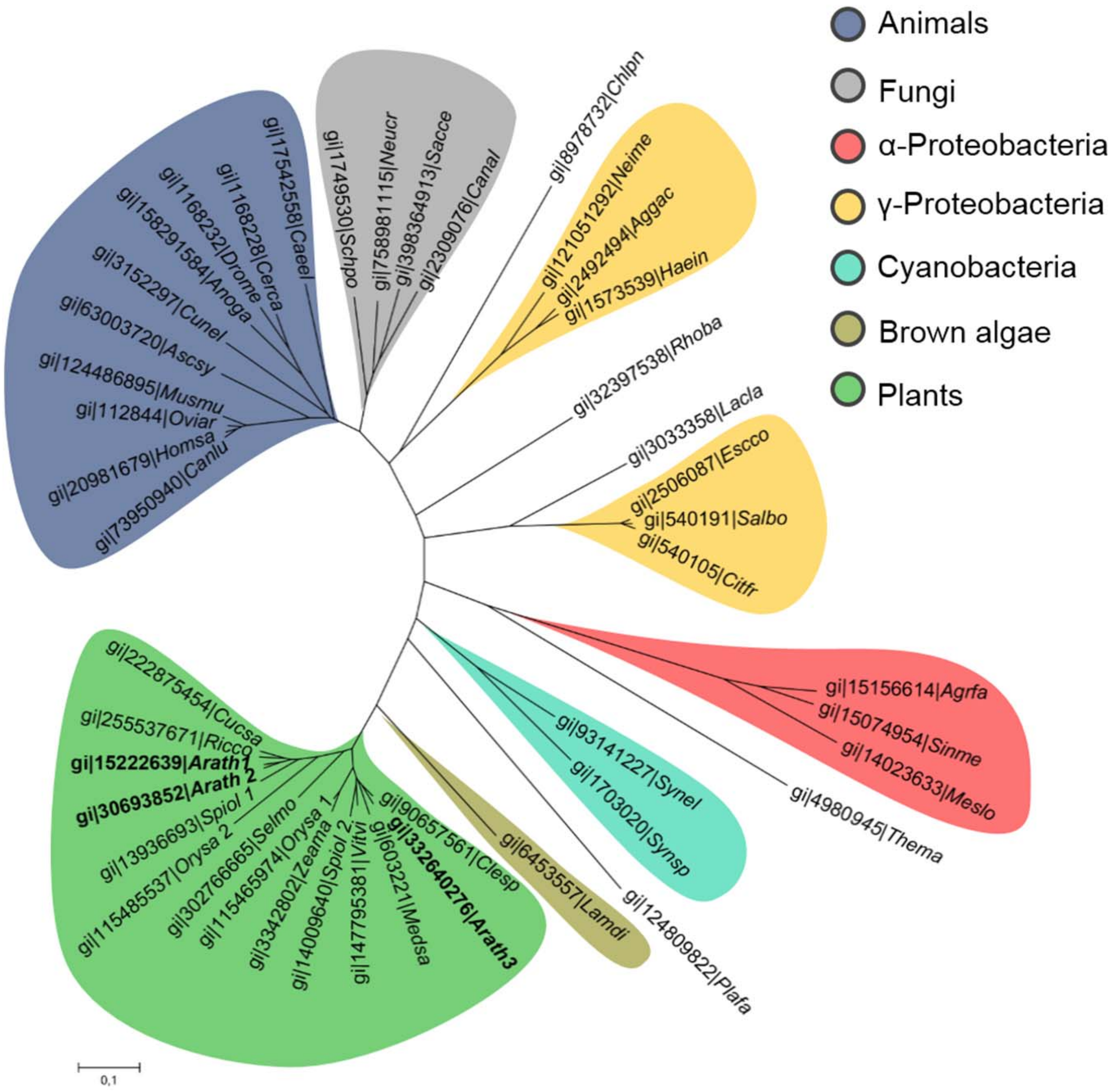

Supplemental Figure 1 Evolutionary relationships of 45 protein sequences of 6PGDH from different taxa. The evolutionary history was inferred using the Neighbor-Joining method. The bootstrap consensus tree inferred from 1000 replicates is taken to represent the evolutionary history of the taxa analyzed. Branches corresponding to partitions reproduced in less than 50% bootstrap replicates are collapsed. The evolutionary distances were computed using the Poisson correction method and are in the units of the number of amino acid substitutions per site. The rate variation among sites was modeled with a gamma distribution (shape parameter = 1). The analysis involved 45 amino acid sequences. All positions containing gaps and missing data were eliminated. There were a total of 376 positions in the final dataset. Evolutionary analyses were conducted in MEGA6 [33].

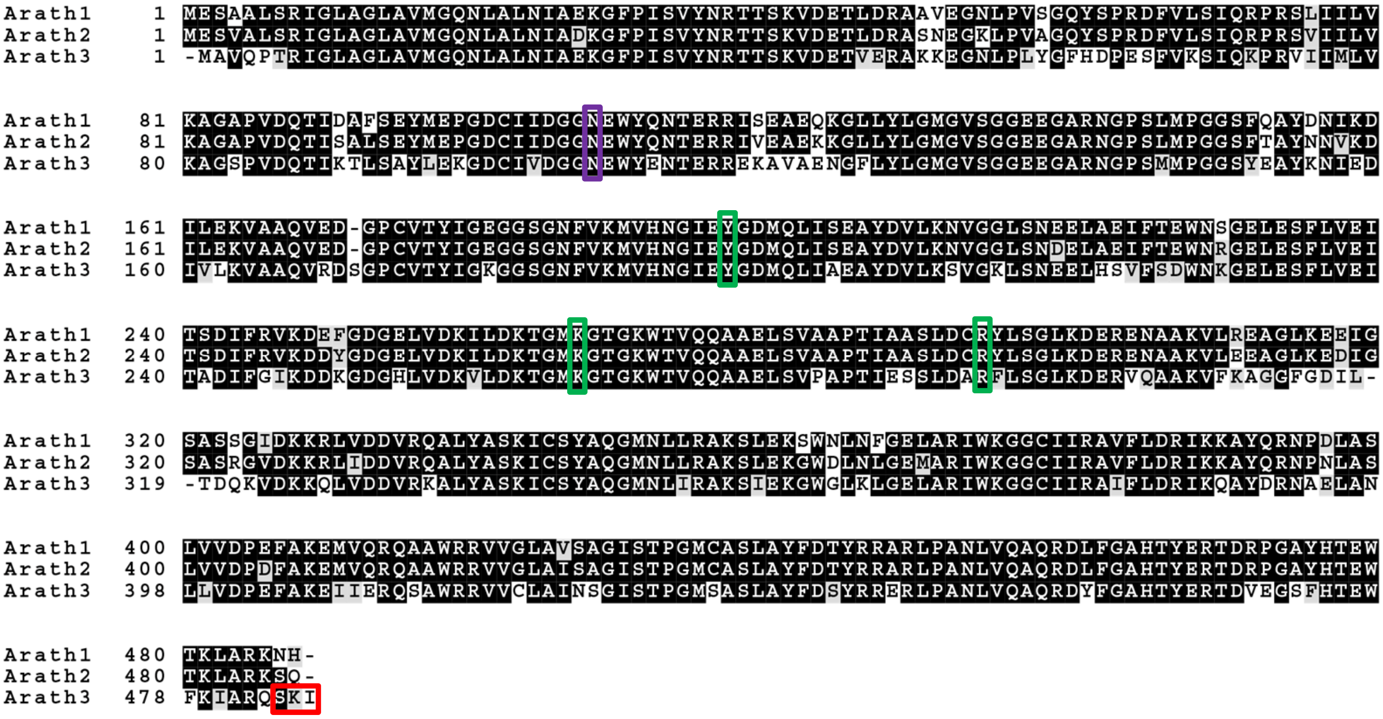

Supplemental Figure 2. Alignment of the three Arabidopsis 6PGDH protein sequences. Sequences corresponding to the three 6PGDH isozymes of *Arabidopsis thaliana* were aligned used ClustalW and edited using BoxShade 3.2. The NADP bind site is marked by a purple box line, the substrate (6PG) binding sites are marked by a green box and the signal sequence for the peroxisome (PTS1) by a solid red line box according to UniProt. Identical residues are indicated by white letters on a black background, whereas similar residues are indicated by black letters on a grey background. Dashes represent gaps to facilitate the alignment..

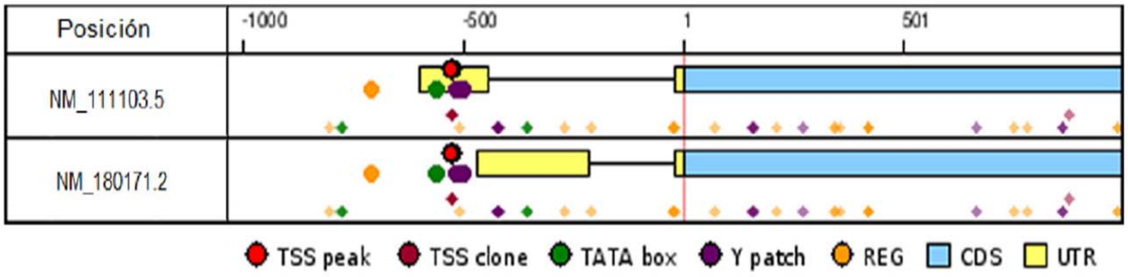

Supplemental Figura 3. Location of the regulatory elements of the promoter (PlantPromoterDB) of peroxisomal 6PGDH in *Arabidopsis thaliana* in the two possible mRNAs designated as NM_111103.5 and NM_180171.2. TSS, transcription start site. TATA box, an octamer group related to TATA box. Y patch, an octamer group of the pyrimidine (Y) patch. REG, regulatory element group, an octamer group related to cis regulatory elements. CDS, protein coding region. UTR, unstraslational region.
